# Knowledge about safe motherhood and HIV/AIDS among school pupils in a rural area in Tanzania

**DOI:** 10.1186/1471-2393-7-5

**Published:** 2007-04-24

**Authors:** Declare L Mushi, Rose M Mpembeni, Albrecht Jahn

**Affiliations:** 1 Department of Tropical Hygiene and Public Health, Ruprecht-Karls-University, Im Neunheimer Feld 324, 69120 Heidelberg, Germany; 2 Department of Epidemiology and Biostatistics, School of Public Health and Social Sciences, Muhimbili University College of Health Sciences, P.O Box 65015, Dar es Salaam, Tanzania; 3 Department of Tropical Hygiene and Public Health, Ruprecht-Karls-University, Im Neunheimer Feld 324, 69120 Heidelberg, Germany

## Abstract

**Background:**

The majority of adolescents in Africa experience pregnancy, childbirth and enter motherhood without adequate information about maternal health issues. Information about these issues could help them reduce their pregnancy related health risks. Existing studies have concentrated on adolescents' knowledge of other areas of reproductive health, but little is known about their awareness and knowledge of safe motherhood issues. We sought to bridge this gap by assessing the knowledge of school pupils regarding safe motherhood in Mtwara Region, Tanzania.

**Methods:**

We used qualitative and quantitative descriptive methods to assess school pupils' knowledge of safe motherhood and HIV/AIDS in pregnancy. An anonymous questionnaire was used to assess the knowledge of 135 pupils ranging in age from 9 to 17 years. The pupils were randomly selected from 3 primary schools. Underlying beliefs and attitudes were assessed through focus group interviews with 35 school children. Key informant interviews were conducted with six schoolteachers, two community leaders, and two health staffs.

**Results:**

Knowledge about safe motherhood and other related aspects was generally low. While 67% of pupils could not mention the age at which a girl may be able to conceive, 80% reported it is safe for a girl to be married before she reaches 18 years. Strikingly, many school pupils believed that complications during pregnancy and childbirth are due to non-observance of traditions and taboos during pregnancy. Birth preparedness, important risk factors, danger signs, postpartum care and vertical transmission of HIV/AIDS and its prevention measures were almost unknown to the pupils.

**Conclusion:**

Poor knowledge of safe motherhood issues among school pupils in rural Tanzania is related to lack of effective and coordinated interventions to address reproductive health and motherhood. For long-term and sustained impact, school children must be provided with appropriate safe motherhood information as early as possible through innovative school-based interventions.

## Background

School children's reproductive health has been a major concern of public health in Africa. Women aged 15–19 in Sub-Saharan Africa, have the highest rates of births when compared to the same age grouping in the rest of the world [1-4]. In some African countries, 30–40% of all women experience motherhood before the age of 18 [5]. Early pregnancy and motherhood is associated with an excess risk of maternal morbidity and mortality and adds to other reproductive health problems such as sexually transmitted infections (STIs), HIV/AIDS and poverty [6-8].

In Tanzania, the majority of children attending school are engaged in early sexual activities before age 15 [9,10]. Three out of four mothers start child-bearing during their adolescence [11]. Most of these mothers have been exposed to primary education [12]. Thus, primary schools provide a unique opportunity for education and discussion of issues related to reproductive health and safe motherhood in particular.

In spite of countrywide education programs, knowledge on reproductive health and HIV/AIDS among pupils appears to be low [13-15] and little is known about school-children's specific knowledge on safe motherhood. It has been argued that school health programs are weak and existing reproductive health education often excludes aspects of safe motherhood. Most school children receive insufficient information or skills on sexuality until it is too late [16,17]. There is a great unmet need for safe motherhood information among school-children [7,18]. Although adequate knowledge does not necessarily alone influence behavior change, there is a consensus that having correct information is fundamental to behavior change [19].

Previous studies on school-based reproductive health programs have focused much on sexuality, sexually transmitted infections and family planning [9,11,15,20]. To our knowledge there is no published study in Tanzania, which focuses on the school-going children's awareness and knowledge of safe motherhood. This study focuses on this important but neglected aspect in understanding the challenges to safe motherhood in Tanzania.

The objective of the study is to assess what school-attending children know and feel about safe motherhood in order to design effective interventions for them. After all, evidence shows that school settings remain the best place to promote attitude and behavior change [21-24].

We describe school children's knowledge and perception about aspects of safe motherhood in the Mtwara rural district of the Mtwara Region in Southern Tanzania. Findings of this study were incorporated in the ongoing community-based safe motherhood intervention.

## Methods

This is a descriptive study, combining quantitative and qualitative methods. It was part of a larger survey conducted in order to develop an ongoing community-based safe motherhood intervention at Kitaya Division in Mtwara region in Tanzania. Mtwara is one of the poorest coastal regions in Tanzania. Mtwara rural district, where the study took place, has the lowest development and health indicators in the region with a total population of 210,000. The dominant tribe is Makonde. Majorities of population are poor subsistence farmers living on less than US$ 1 per day. Maternal mortality ratio in the district is estimated at 600 per 100,000 live births and infant mortality is high at 136 per 000. Only 36% of deliveries occur in institutions, while 61% of deliveries take place at home with unskilled attendants. Unprotected sex, early sexual activities, early pregnancy, early childbearing and early marriage are common health problems in the Mtwara rural district [25,26]. Only about 46% of population in the district have completed primary school education. Among Makonde, initiation rite is an important practice because it is the time when by boys and girls aged between nine and fourteen years old are being prepared to enter into adulthood. In a one-month long training in the bush, girls and boys are taught about cultural traditions, beliefs, taboos, marriage life and responsible parenthood. Circumcision for boys also takes place during this period. Accurate knowledge about safe motherhood amongst the population is low, and cultural beliefs and practices during pregnancy are common [27]. Over 50% of women give birth to their first baby before the age of 19. School drop-out as a result of pregnancy is a common problem in the district [28].

### Study population

All three schools in four villages of the study area were involved. Out of a total of 1100 pupils in all three schools, a sample of 135 pupils (boys and girls) from standard four to seven were randomly selected for the study. Due to the small number of children in the schools (an average of 366 pupils per school) and the homogeneity of the study population; a sample of 135 pupils, which constituted 12% of all the school children in the area, was considered enough. The median age of the children was 13 while the mean age was 11, ranging from 9 to 17 years. In addition ten key informants (six schoolteachers, two community leaders and two health providers) were interviewed. The study was carried out between June and July 2004. Two research assistants were recruited and trained. Instruments for data collection were pre-tested and adjusted accordingly.

### Data collection

The official permission was obtained from the Mtwara Regional Administrative Officer, the Mtwara Rural District Administrative Officer, the Mahurunga Ward Executive Officer and all village chairpersons from the study area. Permission to interview school children was granted by community leaders and schoolteachers. In a planning meeting, community leaders requested explanation of the kind of questions the children would be asked. After explaining the detail of the issues to be covered and the objectives of the study, they saw it as an important but sensitive study. Only school children who had gone through initiation rites were allowed to participate in the study because culturally they are allowed to be taught and to discuss sexuality issues. The sample constituted 22% of pupils who had undergone through initiation process. Before the interview, individual consents were obtained for each pupil.

The assessment of maternal health knowledge among school children was conducted using a self-administered anonymous questionnaire in the absence of schoolteachers. The knowledge test consisted of multiple choice and true and false questions were used to collect quantitative data. The questions were based on the Tanzanian National Reproductive and Child Health Communication Strategy of 2001–2005 and the National Package of Essential Reproductive and Child Health Intervention in Tanzania [29]. In order to get even representation, names of the children were separately listed, and then 135 respondents (45 from each school) were randomly selected to participate in the knowledge test. Fifty-eight girls and 77 boys aged between nine and seventeen years old were recruited. The larger number of boys reflects the higher proportion of boys in school.

Focus Group Interviews (FGIs) and Key Informant Interviews (KIIs) were used to collect in-depth qualitative information. Information collected focused on issues of sexuality, pregnancy, pregnancy complications, danger signs, birth preparedness, delivery, HIV/AIDS, culture and beliefs in pregnancy. FGIs involved school children while KIIs involved schoolteachers and selected community leaders.

From the sample of 135 pupils a total of 39 pupils were purposively selected to participate in the FGIs. Each FGI took approximately 90 minutes. We interviewed girls and boys separately in the absence of schoolteachers. Information was manually recorded during the group interviews. Two FGIs were conducted in each school, altogether six groups. The number of participants in each FGI was 6 girls and 7 boys. More respondents were drawn from standard five and seven with the assumption that, being older they would offer rich information. Other criteria for selection of respondents were as follows: she/he had to be above nine years of age, the individual consented to participate, was able to read and write, was free to communicate and had undergone the traditional initiation process (and was hence culturally allowed to discuss reproductive health issues). Information was manually recorded by the moderators and the direct quotations of the important statements and phrases were immediately recorded.

Interviews were conducted with six teachers, two from each of the three schools, comprising the head-teacher and health teacher. The interview focused on their experiences regarding reproductive health problems among pupils, school health programs, coverage of syllabus, barriers in teaching reproductive and maternal health and their recommendations for improvement. Purposive sampling was used to select four key informants who were considered to be knowledgeable on formal and informal health sectors, traditions and practices. Information collected was cross-checked with schoolteachers and other key people.

All questions for the knowledge questionnaire and FGI were developed in the light of internationally acceptable safe motherhood messages and according to the key health messages disseminated by the Ministry of Health through the Tanzanian National Reproductive and Child Health Communication Strategy of 2001–2005 [29].

### Data analysis

Quantitative data on school children's knowledge of various reproductive health, safe motherhood and STIs was assessed as bi-variant variable with true and false statements. The ability to correctly circle a true answer in a particular question determined the level of knowledge. Quantitative responses were calculated and translated into percentages. The qualitative data were manually analyzed using Content analysis after categorization into main sub-headings. Qualitative data was then classified and analyzed based on thematic topics. Data validation was checked using triangulation techniques. Triangulation was achieved first by employing qualitative (FGI) and quantitative (KT) methods and secondly by interviewing schoolteachers and key informants.

## Results

### Knowledge of safe motherhood

Although the study was not intended to assess in detail knowledge of reproductive health, some basic questions indicate that knowledge of many aspects of reproductive health and sexuality among pupils was low. For example, concerning signs of puberty, 28% of boys and 14.2% of girls could not cite any sign. Only 44% of boys and 33.3% of girls were able to mention three or more signs of puberty such as menstruation, sexual desire, hair growing in private parts and ejaculation. There was no marked difference in knowledge between children from lower and higher classes. During group interview it was learned that pupils felt shy and feared to discuss sexuality, but boys felt freer than girls. One girl in a FGI said,

"Naogopa kusema matusi" which means "I fear to talk about immoral things"

In the knowledge test many girls, especially from lower classes, did not answer some questions which required writing the signs of puberty or reproductive organs. Later during the FGIs we found that they knew the answers but felt shy to write them.

The results of the knowledge test showed that only 33% of pupils could accurately identify the age at which a woman can conceive. Out of 90 (67%) who couldn't mention age at which a woman can conceive, 49 (54.4%) proposed eight to ten years and 41 (46%) cited over twenty years. There was very little difference between boys and girls; only 40% of the boys and 33.3% of the girls got the question right.

Regarding the duration for pregnancy, a total of 104 (77.3%) of the respondents were able to correctly answer nine months, while others suggested longer periods any where from 10 to 20 months. In this question, the girls' knowledge was found to be slightly better (81%) compared to the boys (76%).

### Maternal risk behavior and etiology of pregnancy complications

School children are not well-informed about risk behaviors. For example, when asked to list three risky maternal practices, only 37% mentioned early marriage and early pregnancy and 28% mentioned lack of child spacing. In the FGIs, in discussing the consequences of early marriage, the majority mentioned failure to continue with studies or inability to take care of children or family. After further probing on risk practices very few mentioned heavy work, and unsafe sex. Abortion, smoking, alcoholism and violence were never mentioned. Nothing was known about post-partum complications or care. The few pregnancy complications and danger signs, which were known and repeatedly mentioned in all FGIs, were difficulties in delivery and anaemia.

On birth preparedness, the majority of pupils were aware and they mentioned the need to save money, for food and transport. Other preparedness issues such as early antenatal care (ANC) visits, identification of skilled birth attendants, birth plan and the identification of blood donors were not mentioned. A question regarding where they would prefer to deliver in the future, the whole group loudly replied "*Kituo cha Afya*" (health center). When asked why, they said, "because there is reliable medical help"

### Pregnancy, traditional beliefs and practices

It is very interesting that school children, especially those who have gone through initiation rites, are well informed about traditions related to pregnancy and childbirth. Strikingly, as seen in Table 1, the majority of pupils (63.4%) often associated pregnancy complications, difficult labour and negative pregnancy outcome with non-observance of traditional rules and taboos. During a FGI a standard seven girl said,

**Table 1:** Knowledge Test: Children's knowledge on maternal health aspects

	Percentage of correct answers*		
**Statements**	**Boys (n = 77)**	**Girls (n = 58)**	**Total (n = 135)**

School children can be infected with HIV/AIDS (True)	88%	91%	89.5%
Girls are not advised to be married before they reach 18 years old (True)	88%	71.4%	79.7%
It is not possible to plan how many children one should have (False)	72%	62%	67%
It is advised to have child before reaching age 18 (False)	62.3%	48%	55.2%
HIV/AIDS can be transmitted from mother to child (True)	52%	56%	54%
For safe delivery in Tanzania, women are advised to deliver at home (False)	44%	42.9%	43.5%
It is very important for women to observe all traditions for a safe delivery (False)	48%	23.8%	35.9%

*Answers were judged according to Tanzanian reproductive health and safe motherhood massages.

*"My mother told me that most women in our village get difficult labour because one spouse or both had sex with another person during pregnancy"*.

Others supported this statement. From the KIIs, it was noted that this belief is popular among community members. Another belief mentioned was, if a husband is traveling he should not inform his pregnant wife who is expecting to deliver soon. If he does, the child will refuse to come out until the father reaches the destination. Several taboos that restrict pregnant women from eating certain types of food were also mentioned. Few pupils doubted these beliefs and practices and were eager to get more clarifications.

### Knowledge on STI and HIV/AIDS in pregnancy

When asked to state any three STIs that they know, 23 out of 77 boys (30%) and 14 out of 58 girls (24.1%) mentioned only one STI respectively. The majority, over 60% of all respondents were able to mention more than two STIs; only 27% were able to mention three. Most pupils mentioned syphilis, gonorrhea and HIV/AIDS, and some included schitosomiasis. FGIs revealed that, the majority was aware that STIs can be transmitted through sexual contact, but no one mentioned the possibility of transmission during childbirth.

Almost all school children have heard about HIV/AIDS. However, very few reported to have seen anyone suffering from AIDS. When asked to list five ways HIV is transmitted, only 50% were able to mention three or more ways and 24% stated only one way. Generally, pupils from older classes were more knowledgeable than those from younger classes. We noted some misconceptions about the disease. When answering the question whether they think school children can be infected with HIV, in two FGIs, the statement was made "*We are still very young, we cannot be infected" *and themajority in the group seemed to support this statement. In some of the groups HIV/AIDS was seen as an urban problem.

Knowledge about the signs of AIDS was found to be higher; seventy six percent the school children stated more than three signs. Again more boys (84%) were able to volunteer more than three HIV/AIDS signs compared to girls (66.6%). Commonly mentioned signs were loss of weight, prolonged coughing and diarrhea. About 41% were able to mention three or more ways of HIV/AIDS prevention. The commonly cited prevention measure was abstinence. A small proportion of children, mainly being boys, mentioned condom use. In FGIs, children mentioned traditional night dances, alcoholism, unsafe sex, and urban life to be major influencing factors for HIV/AIDS transmission.

The majority of children, 126 (93.3%) are aware that the virus can be transmitted from mother to child but the ways to prevent transmission from mother to child was almost unknown to the majority of the pupils. Quite a large percentage of children, 103 (76.3%) mentioned avoidance of extra-marital sex with high risk groups like prostitutes as the major way of preventing transmission from mother to child. Out of 135 respondents, only 24 (17.8%) were aware that HIV/AIDS can be transmitted during birth and through breastfeeding. In most aspects regarding HIV/AIDS transmission, boys overall were slightly more knowledgeable than girls.

With regard to the sources of health information, over 68% of school children said their main sources of reproductive health and safe motherhood information are friends, out of school youths, parents and schoolteachers. Only 17% mentioned media and about 11% mentioned health staffs and village health workers.

## Discussion

This study confirms a lack of basic knowledge on safe motherhood and related reproductive health issues, including HIV/AIDS [4,30,31]. Beyond that, it provides new insight into the pupils' knowledge of pregnancy and childbirth, which includes the following: -

(1) The limited knowledge and awareness of key maternal issues such as, knowledge of normal pregnancy duration, danger signs and maternal risk behaviours.

(2) The presence of considerable information on cultural traditions and practices related to pregnancy and childbirth.

(3) Hardly any knowledge about vertical HIV/AIDS transmission from mother to child and its prevention measures.

The discussion will focus on these three observations.

The key findings reveal that the majority of adolescents and particularly girls experience pregnancy and motherhood at an early age, without prior information about safe motherhood. Generally, the problem can be associated with the overall weakness of school health and reproductive education programs [17].

### Knowledge about safe motherhood

Issues such as pregnancy complications, danger signs, antenatal and postnatal care, birth preparedness, the importance of a skilled attendant during delivery, and post-abortion care were almost unknown to the pupils because they are never taught in school. The components of birth preparedness such as saving money, food and arrangement of transport were known to most children because these are common problems in their community. In FGIs most respondents were in favour of birth preparedness plan and institutional delivery. This is a good sign showing that early and effective interventions can have greater potential to influence and promote utilization of skilled birth attendants for safer motherhood. Interestingly, boys were found to be more knowledgeable than girls in many aspects of reproductive health and safe motherhood. This suggests that boys have more access to health information than girls do, or there is more information sharing among boys and between boys and adults than there is among girls or between girls and adults. In this regard, if boys are targeted earlier they can be potential partners in promoting reproductive health and safe motherhood. Generally, the above problems appear to be associated with an overall weakness of school health education in general, and reproductive health education in particular [17]. Schoolteachers and key informants expressed the urgency for culturally sensitive reproductive health education in schools especially for children who are above 10 years of age.

### Pregnancy, traditional beliefs and practices

School children, especially those, who have gone through initiation rites, are well informed about cultural traditions related to pregnancy and childbirth. The majority of respondents associated pregnancy complications with non-observance of traditions. This is not surprising because previous studies in the same region found similar beliefs and traditions are deep-rooted amongst populations [25,27]. Boys and girls learn about traditions through peer-effect, the socialization process at home and in the community and during initiation rites. However, fear and shyness are related to cultural taboos of discussing sexuality. Probing about the contents and skills taught during traditional rites, led the researcher to question whether these rites can impart the necessary knowledge to promote safe motherhood. School children mentioned their main sources of health information to be friends, out of school youth, parents and schoolteachers. This implies that effective and long term programs such as peer education, school and community-based safe motherhood interventions can positively improve knowledge, effect behaviour change, and increase the utilization of maternal services, hence improving maternal outcome [21,22].

### Knowledge on STI and HIV/AIDS

Despite the increased amount of HIV/AIDS education in the region and in schools, misconceptions were noted about routes of infection and ways to prevention infection. These results are supported by other findings elsewhere in the country [4,15,32]. With regard to HIV/AIDS in pregnancy, most pupils did not indicate knowledge about HIV transmission from mother to child or ways to prevent it but reiterated general health messages such as "to avoid sex with prostitutes". The knowledge gap is evident; the dominant thinking is that one should avoid sex with high-risk individuals, rather than to avoid sex entirely or practicing safe sex. This reflects the fact that in this area pre-marital and extra-marital sex are culturally expected and "encouraged" by traditions and reinforced with existing misleading information on how to prevent infection [26]. FGIs revealed the general perception was that AIDS is an urban problem, rural women are safer than urban women, children are much safer and sex with prostitutes is risky.

In Tanzania, STI and HIV/AIDS lessons are incorporated in the school curriculum. But very few schoolteachers, particularly in rural settings, have been trained to teach these health topics. Schoolteachers are not well prepared and trained on participatory communication methods to help children learn better [17]. Interviews with head-teachers revealed that in all three schools, only 2 out of 22 schoolteachers had attended a one-day HIV/AIDS seminar between the year 2002 and June 2004. Difficulty in communicating about sexuality to pupils was reported by the head of schools. Two main communication barriers were identified. Firstly, lack of skills on how to teach sexuality and reproductive health. Second, resistance from parents because they think children will learn "immoral" things. School-based reproductive health programs can be effective if schoolteachers are well prepared and the community is involved in designing interventions. In Tanzania where nearly half of all deaths of women of reproductive age are caused by HIV/AIDS, early information on HIV/AIDS transmission and prevention from mother to child is crucial in the ongoing effort to reduce maternal mortality.

## Conclusion

To our knowledge no studies in the country have addressed school children's knowledge and perception on safe motherhood. This study has brought to light that school children in rural areas lack information and awareness on many aspects of safe motherhood due to ineffective school health programs, reproductive and maternal health interventions and ineffective traditional initiation rites which do not prepare adolescents on sexuality, puberty and safe motherhood. This means that most adolescents, particularly girls enter motherhood and parenthood at an early age without prior information about safe motherhood.

In Tanzania, where three out of four mothers start childbearing before the age of 18, and the majority of this age group pass through the primary school, the need to include safe motherhood in school health curriculum is crucial. School children have the right to appropriate information. From their early teen years, schoolchildren need systematic and large amounts of education on reproductive and maternal health. They need to learn topics such as: the dangers of early marriage and, early pregnancies, unsafe sex, un-spaced pregnancies, abortion and HIV/AIDS prevention from mother to child. Other issues that are of equal importance are pregnancy care, maternal danger signs, childbirth, birth preparedness, post-natal care, complications and utilization of health services. Over time, well-designed school-based reproductive health programs, through peer influence and diffusion process can be beneficial to out-of-school youth and to the whole community. This calls for political commitment, careful planning, adequate resources and coordinated efforts among all partners responsible for the welfare of the adolescent population.

As with all studies, this study had two main limitations. First, the sample size was too small to make statistical generalization of the situation in the Mtwara region. Secondly, since the researchers were outsiders, there is possibility that, school children and particularly girls could not disclose all the information due to shyness, cultural barriers and taboos in discussing about sexuality.

## Competing interests

The author(s) declare that they have no competing interests.

## Authors' contributions

DM designed the study, participated in the data collection, data analysis and drafted the manuscript for publication. RM was involved in the study design, data analysis and preparation of the manuscript. AJ offered scientific advice, inputs and critique during the study design, data collection and analysis and throughout the preparation of the manuscript. All authors read and approved the final manuscript.

## Pre-publication history

The pre-publication history for this paper can be accessed here:


